# Tailorable stimulated Brillouin scattering in nanoscale silicon waveguides

**DOI:** 10.1038/ncomms2943

**Published:** 2013-06-06

**Authors:** Heedeuk Shin, Wenjun Qiu, Robert Jarecki, Jonathan A. Cox, Roy H. Olsson, Andrew Starbuck, Zheng Wang, Peter T. Rakich

**Affiliations:** 1 Sandia National Laboratories, Albuquerque, New Mexico 87185, USA; 2 Department of Physics, Massachusetts Institute of Technology, Cambridge, Massachusetts 02139, USA; 3 Department of Electrical and Computer Engineering, University of Texas at Austin, Austin, Texas 78758, USA; 4 Department of Applied Physics, Yale University, New Haven, Connecticut 06520, USA

## Abstract

Nanoscale modal confinement is known to radically enhance the effect of intrinsic Kerr and Raman nonlinearities within nanophotonic silicon waveguides. By contrast, stimulated Brillouin-scattering nonlinearities, which involve coherent coupling between guided photon and phonon modes, are stifled in conventional nanophotonics, preventing the realization of a host of Brillouin-based signal-processing technologies in silicon. Here we demonstrate stimulated Brillouin scattering in silicon waveguides, for the first time, through a new class of hybrid photonic–phononic waveguides. Tailorable travelling-wave forward-stimulated Brillouin scattering is realized—with over 1,000 times larger nonlinearity than reported in previous systems—yielding strong Brillouin coupling to phonons from 1 to 18 GHz. Experiments show that radiation pressures, produced by subwavelength modal confinement, yield enhancement of Brillouin nonlinearity beyond those of material nonlinearity alone. In addition, such enhanced and wideband coherent phonon emission paves the way towards the hybridization of silicon photonics, microelectromechanical systems and CMOS signal-processing technologies on chip.

## Abstract (web summary)

Exploiting photon–phonon coupling in nanoscale silicon waveguides could enable a host of powerful features in photonic devices. Using a hybrid photonic–phononic waveguide structure, Shin *et al*. show stimulated Brillouin scattering nonlinearities and gain, which offers new on-chip signal-processing abilities.

## Graphical abstract

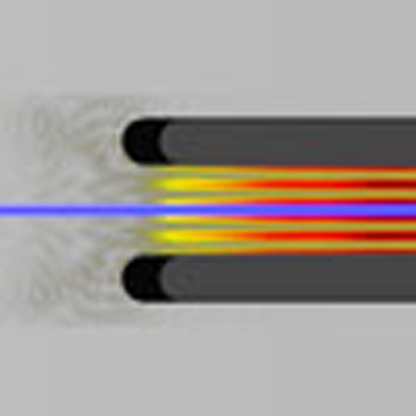

Photon–phonon coupling through guided-wave stimulated Brillouin scattering (SBS) provides a powerful means of realizing tailorable slow light^1^,^2^, radio frequency (RF)–photonic signal processing^3^,^4^, narrow-line-width laser sources^5^,^6^,^7^,^8^,^9^,^10^,^11^, RF–waveform synthesis^12^,^13^,^14^ and optical frequency comb generation^12^,^15^,^16^. Realization of this form of travelling-wave photon–phonon coupling in a silicon-based and CMOS (complementary metal–oxide–semiconductor)-compatible platform could enable high-performance signal-processing applications through nanoscale Brillouin interactions^17^,^18^. Nanoscale modal confinement has been shown to radically enhance non-linear light-matter interactions within silicon waveguides^19^,^20^,^21^,^22^ and in nano-optomechanics^23^,^24^,^25^,^26^. For instance, tight optical confinement in nanoscale silicon waveguides is responsible for greatly enhanced Raman and Kerr non-linearities^19^,^20^,^21^,^22^,^27^, and for new sensing^23^, actuation^23^,^28^,^29^ and transduction^30^ mechanisms based on optical forces within nano-optomechanical systems.

The field of cavity optomechanics has produced a wide variety of systems with enhanced and controllable forms of photon–phonon coupling^25^,^31^,^32^,^33^. Specifically, silicon-based cavity-optomechanical systems^30^,^34^,^35^ have recently enabled powerful new forms of quantum state transfer^34^,^36^, slow light^37^, phonon lasers^38^ and optomechanical ground-state cooling^39^. Such cavity systems exploit resonantly enhanced coupling between discrete photonic and phononic modes. As a fundamental complement to cavity systems, guided-wave Brillouin processes produce coupling between a continuum of photon and phonon modes for a host of wideband (0.1–34 GHz) RF and photonic signal-processing applications^12^,^40^,^41^,^42^,^43^. For example, travelling-wave Brillouin processes have enabled unique schemes for optical pulse compression^13^, pulse and waveform synthesis^12^,^14^,^15^,^44^, coherent frequency comb generation^12^,^15^,^16^, variable bandwidth optical amplifiers^44^,^45^, reconfigurable filters^46^ and coherent beam-combining schemes^47^. Although there are a variety of compelling opportunities for chip-scale Brillouin technologies, to date the observation of Brillouin processes in silicon nanophotonics has proven difficult; strong Brillouin non-linearities require large optical forces and tight confinement of both phonons and photons, conditions that are not met in conventional silicon waveguides^41^.

In this paper, we demonstrate travelling-wave forward SBS nonlinearities and forward SBS gain in silicon waveguides for the first time through a novel class of hybrid photonic–phononic waveguides. Confinement of both photons and phonons is achieved using a Brillouin-active membrane waveguide structure, eliminating the substrate pathway for phonon losses that stifles SBS in conventional silicon-on-insulator waveguides. In contrast to the theoretical work presented in ref. 41, this compound-material waveguide geometry provides independent control of the photonic and phononic waveguide modes and the resulting Brillouin spectra. We show that these degrees of freedom enable the placement of forward SBS resonances between 1 and 18 GHz through phase-matched coupling to ultra-low group velocity-guided acoustic waves. The strength and bandwidth of photon–phonon coupling is quantitatively studied using heterodyne four-wave mixing (FWM) techniques and through direct measurements of Brillouin gain. Experiments reveal that forward SBS non-linearities surpass those generated by the intrinsic Raman and Kerr non-linearities in silicon, corresponding to a Brillouin non-linear coefficient that is more than 1,000 times greater than prior demonstrations of forward SBS^12^. The magnitude and frequency dependence of the Brillouin coupling show excellent agreement with multi-physics models, revealing that a coherent combination of electrostrictive (ES) forces^48^ and boundary-induced radiation pressures^28^,^49^ is responsible for the enhancement of Brillouin interactions within these nanoscale waveguides. The emergence of strong radiation-pressure-mediated Brillouin coupling is noteworthy, as it represents a new regime of Brillouin non-linearity arising from strong boundary interactions in subwavelength limit of confinement^41^,^48^,^49^. Detailed analysis of the waveguide nonlinear responses also reveals that a coherent interference between Kerr and Brillouin non-linearities either cancel or enhance the total third-order nonlinearity at individual frequencies.

## Results

### A Brillouin-active membrane waveguide

**Figure 1:**
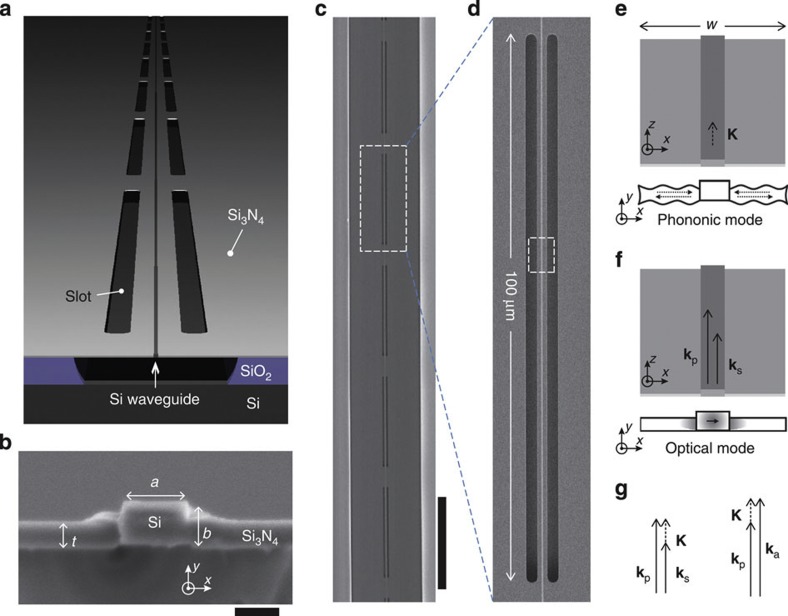
Hybrid photonic–phononic waveguide enabling independent control of the optical and phonon modes. (**a**) Diagram showing anatomy of the Brillouin-active membrane waveguide (BAM waveguide). (**b**) High-resolution SEM cross-section of the silicon waveguide core with width *a*=313 nm and height *b*=194 nm within the silicon nitride membrane with thickness *t*=124 nm. Scale bar, 200 nm. (**c**) Top-down SEM image of a portion of the BAM waveguide displaying a series of five identical membrane-suspended regions. The scale bar is 100 μm. (**d**) Magnified view of single suspended region with *w*=3.8 μm. (**e**,**f**) e and f illustrate propagation of the guided elastic and optical modes respectively within the highlighted section of **d**. (**g**) Vector phase matching diagrams for Stokes and anti-Stokes forward-stimulated Brillouin scattering; **k**_p_, **k**_s_ and **k**_a_ represent the optical pump, Stokes, and anti-Stokes wave-vectors; **K** is the phonon wave-vector.

Figure 1a is a schematic showing the anatomy of the fabricated Brillouin-active membrane waveguide (or BAM waveguide). The BAM waveguide under study is seen in the top-down scanning electron microscopic (SEM) images of Fig. 1c, showing a silicon nanophotonic waveguide embedded within the centre of a series of five suspended phononic waveguide segments. The SEM image in Fig. 1b shows a cross-section of the waveguide core at one point along its length. The BAM waveguide consists of a nanoscale (313 × 194 nm) silicon waveguide embedded in a tensile silicon nitride membrane (thickness 124 nm). As illustrated in Fig. 1e, total internal reflection between silicon (*n*=3.5) and silicon nitride (*n*=2.0) tightly confines the optical mode to the silicon waveguide core, here the patterned silicon nitride membrane acts to guide the generated phonons. This compound-material device geometry provides independent control of the photonic and phononic waveguide dispersion, allowing the phonon modes to be shaped separately from the optical forces within the core of the silicon waveguide.

### Phase-matched coupling via forward SBS

**Figure 2:**
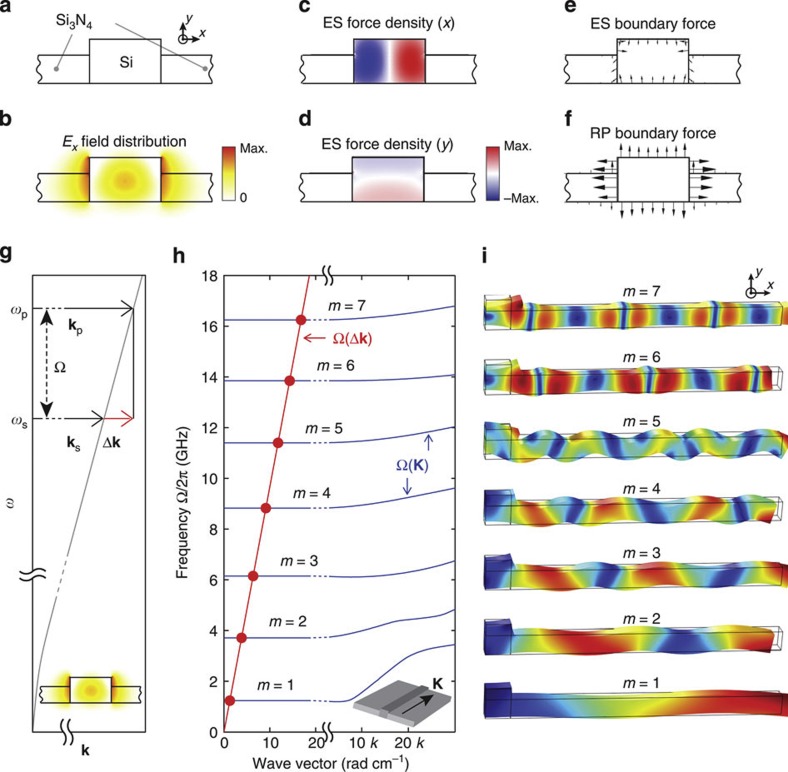
Simulations of hybrid photonic–phononic waveguide. (**a**,**b**) The waveguide cross-section and the computed *E*_*x*_ field profile of the optical mode. (**c–f**) Computed force distributions associated with the guided optical mode. (**c**,**d**) The *x* and *y* components of the ES force densities generated within silicon, respectively. (**e**,**f**) ES- and radiation pressure (RP)-induced boundary forces, respectively, produced by the optical mode. (**g**) Sketch showing the optical wave vector mismatch, Δ**k**, produced by the dispersion of the optical waveguide mode, *ω*(**k**), as the pump and stokes waves are detuned. (**h**) Dispersion curves showing phonon frequency versus longitudinal wave vector of the Brillouin-active phonon modes for a *w*=3.8 μm BAM waveguide. A break in the scale of the *x* axis is shown. For small wave vectors (left), the phase-matched phonons (circles) are identified by the intersection between the optical wave vector mismatch (red) and the Brillouin-active phonon modes (blue). (**i**) Displacement fields of each of the phase-matched guided-wave phonon modes identified in **h**. Each mode is labelled (*m*=1,2…7) according to the mode index in **h**. The right half of each displacement field is shown as the symmetric force distributions of **c**–**f** only permit coupling to phonons with symmetric displacement fields about the waveguide core. Periodic boundary conditions were applied to the *z*-normal faces of the simulation domains in **i** to capture the travelling-wave nature of these guided phonons at the phase-matched **K**-values identified in **h**.

This paper examines intramodal forward SBS through Brillouin coupling between guided transverse-electric-like optical modes of the type seen in Fig. 2b. The computed ES force densities (Fig. 2c–e) and radiation pressure-induced force densities (Fig. 2f) that mediate Brillouin coupling are shown in Fig. 2. Through forward SBS, copropagating pump and Stokes waves of frequencies *ω*_p_ and *ω*_s_, respectively, couple through parametrically generated acoustic phonons of difference frequency Ω=*ω*_p_−*ω*_s_. Momentum conservation requires that **k**(*ω*_p_)=**K**(Ω)+**k**(*ω*_s_), where **k**(*ω*) is the optical dispersion relation, and **K**(Ω) is the phonon-dispersion relation. Thus, strong photon–phonon coupling is mediated by the set of phonons, {Ω_**i**_}, whose dispersion relations satisfy the phase-matching condition Δ**k**(Ω)=**k**(*ω*_p_)−**k**(*ω*_p_−Ω)=**K**(Ω). Vector representation of this phase-matching condition is seen in Fig. 1g.

The computed dispersion relations of the Brillouin-active phonon modes guided by a BAM waveguide of width *w*=3.8 μm are seen in Fig. 2h. For further details of the simulation methods, see Supplementary Note 1. Only the phonon modes that exhibit strong Brillouin coupling through good overlap between the elastic displacement fields and the optical force distributions are shown^41^,^50^. The optical wave vector mismatch, Δ**k**(Ω), is plotted (red) atop the phononic dispersion relation, **K**(Ω), as seen in Fig. 2h. The points of intersection between these curves (seen as circles) identify the Ω- and **K**-values of the phase-matched phonon modes. Numerous phase-matched phonon modes are seen with evenly spaced frequencies spanning 1–16 GHz, and corresponding values of |**K**| between 1.3 and 17 rad cm^−1^. The BAM waveguide consists of a periodic-array Brillouin-active suspended regions, as seen by the SEM micrograph of Fig. 1c. As the spatial period of the waveguide modulation (125 μm) is more than 20 times smaller than the longitudinal period of the guided phonon wave vector (2π/|**K**|), the phase-matching conditions are unaffected.

On the basis of the phase-matching condition and the relation 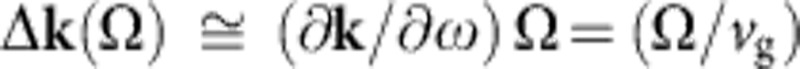, only guided phonons with phase velocities matching the group velocity of light (*v*_g_) produce resonant coupling through forward SBS. This is because the interference between the copropagating pump and Stokes waves yields modulated energy density and force density distributions that propagate along the waveguide at the group velocity (*v*_g_) of light. As this travelling-force distribution drives photon–phonon coupling, only phonons with phase velocities (Ω/**K**) matching the group velocity (*v*_g_) of light can produce efficient coupling (seen as circles in Fig. 2h). These ultra-high phase-velocity-guided phonon modes (~10^8^ m s^−1^) have corresponding guided phonon group velocities (*∂*Ω/*∂***K**) that are exceedingly slow (~1 m s^−1^), but are non-zero.

The displacement field associated with each of the phase-matched Brillouin-active guided wave modes is shown in Fig. 2i. Periodic boundary conditions are applied to the *z*-normal faces of this simulation domain to compute the displacement fields of the phase-matched phonon modes in Fig. 2i and the corresponding phonon-dispersion curves in Fig. 2h. Although these guided elastic modes exhibit some flexural character, the vast majority of the modal potential energy is ascribed to in-plane (*x*–*z*) elastic compression. The compressive character of these slow group velocity-guided modes is most clearly seen in the high frequency limit, as demonstrated by the displacement fields of the *m*=6 and *m*=7 modes in Fig. 2i. For small **K**-values, the *z*-component of the phonon displacement field becomes much smaller than the *x*-component. Moreover, in the limiting case where the waveguide possesses vertical symmetry (that is, for *t*=*b*), these Brillouin-active modes exactly converge to symmetric Lamb waves with nearly identical dispersion curves to those seen in Fig. 2g. Hence, these waves are classified as symmetric Lamb waves, producing equal frequency spacing of the phase-matched Brillouin modes seen in Fig. 2h. For further details concerning mode classification and elastic wave simulation, see Supplementary Note 1.

The waveguides under study have a total length of *L*=4.9 mm, with 26 Brillouin-active membrane-suspended regions spanning a 3.3-mm section of this length. This 3.3 mm Brillouin-active device length coincides with a total non-linear phase mismatch, |**Δk**|·*L*, of between 0.45 and 5.7 radian for the range of Brillouin-active modes identified in Fig. 2. Previous nano-optomechanical waveguide systems of shorter length scales have been accurately treated as lumped-element systems^23^,^24^. However, this travelling-wave device produces a significant non-linear phase mismatch along its length. Thus, it is necessary to treat this system as a phase-matched travelling-wave process to describe coherent addition of non-linearities along the entire length of the waveguide as in ref. 12.

### Non-linear Brillouin spectroscopy

Through experiments, an array of BAM waveguides are studied with waveguide widths, *w*, between 0.8 and 3.8 μm, producing a wide range of Brillouin resonances between 1 and 18 GHz. As the optical group velocity, *v*_g_, changes by only a few per cent over a 30-nm wavelength range, the optical phase mismatch (with values, 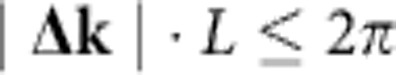) changes to negligible degree over an appreciable wavelength range. As a consequence, the same guided phonon can be excited by continuum of different wavelengths within the BAM waveguide despite the fact that it is a phase-matched non-linear process^12^,^18^. This property of the system allows pump and probe waves of disparate wavelengths to couple to each other through the Brillouin-active modes of a single device. To obtain large Brillouin non-linearities, a serial array of 26 suspended regions were fabricated along each waveguide, yielding a Brillouin-active length of 3.3 mm out of a total 4.9 mm device length. Note that each suspended waveguide section is separated by a 25-μm anchored (or unsuspended) region.

**Figure 3:**
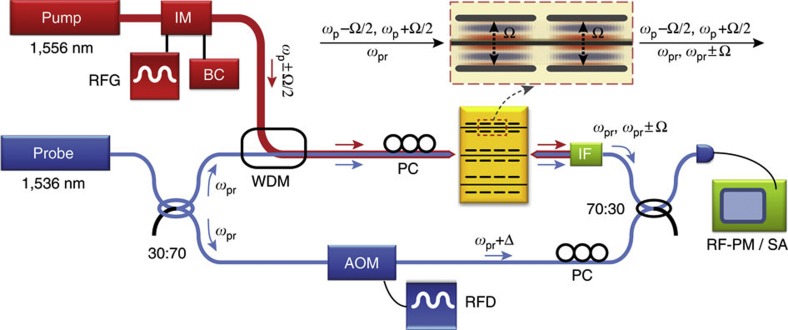
Heterodyne two-colour pump–probe apparatus. Schematic diagram of the apparatus used to measure the Brillouin non-linearity of the BAM waveguide. The non-linearly induced phase changes imparted to the probe beam (1,536 nm) by the waveguide device are detected using heterodyne interferometer, whereas a modulated pump beam (1,556 nm) produces driven excitation of the Brillouin-active phonon modes. Components of the apparatus are labelled as follows: ISO, isolator; PC, polarization controller; IM, intensity modulator; RFG, RF generator; BC, bias controller; WDM, wavelength division multiplexer; AOM, acousto-optic modulator; RFD, RF driver; IF, interference filter; SA, spectrum analyser; RF-PM, RF power meter.

Experimental studies of Brillouin non-linearity were performed with the heterodyne FWM apparatus, seen in Fig. 3, yielding direct measurement of the third-order non-linear susceptibility. Through FWM experiments, modulated pump (1,556 nm) and continuous-wave probe (1,536 nm) signals are injected into the device in a measurement configuration similar to that used in refs 23, 51. The modulated pump drives the excitation of Brillouin-active phonons over a wide range of frequencies as the pump modulation frequency is swept. The non-linear response of the device is then analysed by heterodyne measurement of optical tones imprinted on the disparate probe wavelength due to a coherent combination of the Brillouin and third-order electronic non-linear susceptibilities (that is, through FWM). These sidebands are then analysed as distinct RF tones through heterodyne interferometry. In contrast to the approach of refs 23, 51, this heterodyne approach allows the Stokes and anti-Stokes signatures to be resolved separately. For further details, see the Methods section.

**Figure 4:**
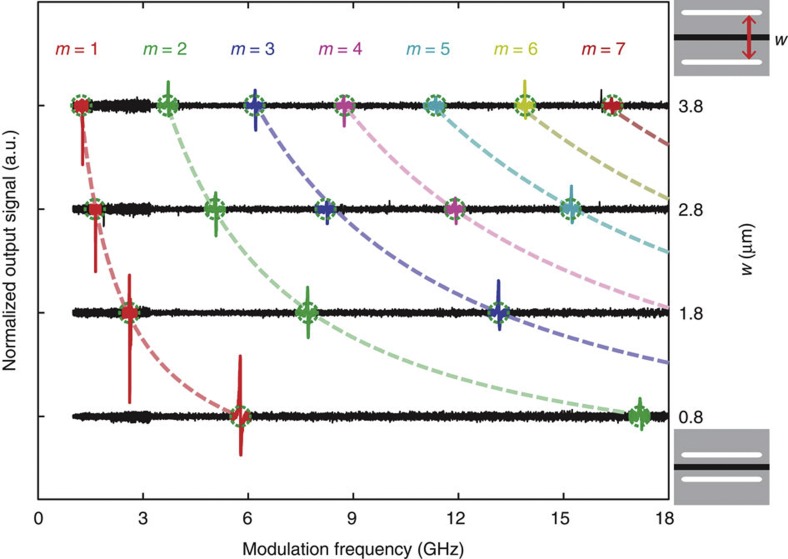
Spectra of non-linear Brillouin spectra obtained through heterodyne FWM measurements. The resonant Brillouin signatures produced by several BAM waveguides are displayed for waveguide dimensions *w*=[0.8, 1.8, 2.8, 3.8] μm. Each trace is obtained by normalizing the signal produced by each Brillouin-active waveguide to that of an identical reference silicon waveguide (one that is not Brillouin active) under identical experimental conditions. The dashed blue circles shown atop the experimental traces show the numerically computed frequencies of each Brillouin-active phonon mode as the waveguide dimension, *w*, varies. The upper right inset is a schematic geometry of the BAM waveguide. Each waveguide has the resonant modes labelled by a mode index, *m*, as in Fig. 2.

As seen from Fig. 4, clear signatures showing the non-linear Brillouin response were observed by measuring the intensity of non-linearly induced sidebands imprinted on the probe, as the pump modulation frequency was swept from 1 to 18 GHz. The spectra in Fig. 4 were obtained by integrating the RF power produced by heterodyne detection of the probe signal (including both Stokes and anti-Stokes sidebands) over a discrete set of high-frequency RF bands using RF filters. To remove the frequency dependence of the detection system and to more clearly exhibit the sharp Brillouin resonances, the spectra in Fig. 4 were normalized to those of an identical optical waveguide without a Brillouin-active region. Each waveguide produces a series of regularly spaced Brillouin resonances analogous to those identified in Fig. 2. Because of the spatial symmetry of the optical force distribution, only phonon modes with even displacement symmetry with respect to the waveguide core produce efficient Brillouin coupling. The different resonant signatures are colour coded (red, green, blue and so on) to indicate the mode order (1st, 2nd, 3rd and so on) of each phononic resonance as the Brillouin spectrum shifts with waveguide dimension. All 17 experimentally observed resonances between 1 and 18 GHz showed good agreement with simulated mode frequencies over the range of device dimensions. The simulated modal frequencies are displayed as dashed curves atop the experimental data. Figure 4 reveals that a variation of the cavity dimension allows precise placement of Brillouin resonances at virtually any frequency from 1 to 18 GHz, for an unprecedented degree of non-linear tailorability. For example, the *m*=2 resonance (green) is shifted from 3.7 to 17 GHz, as the cavity dimension, *w*, varied from 3.8 to 0.8 μm. Although the bandwidth limitations of our apparatus did not permit measurements beyond 18 GHz, strong Brillouin resonances are expected at 25 GHz and higher frequencies.

### Quantitative analysis of the Brillouin non-linearity

**Figure 5:**
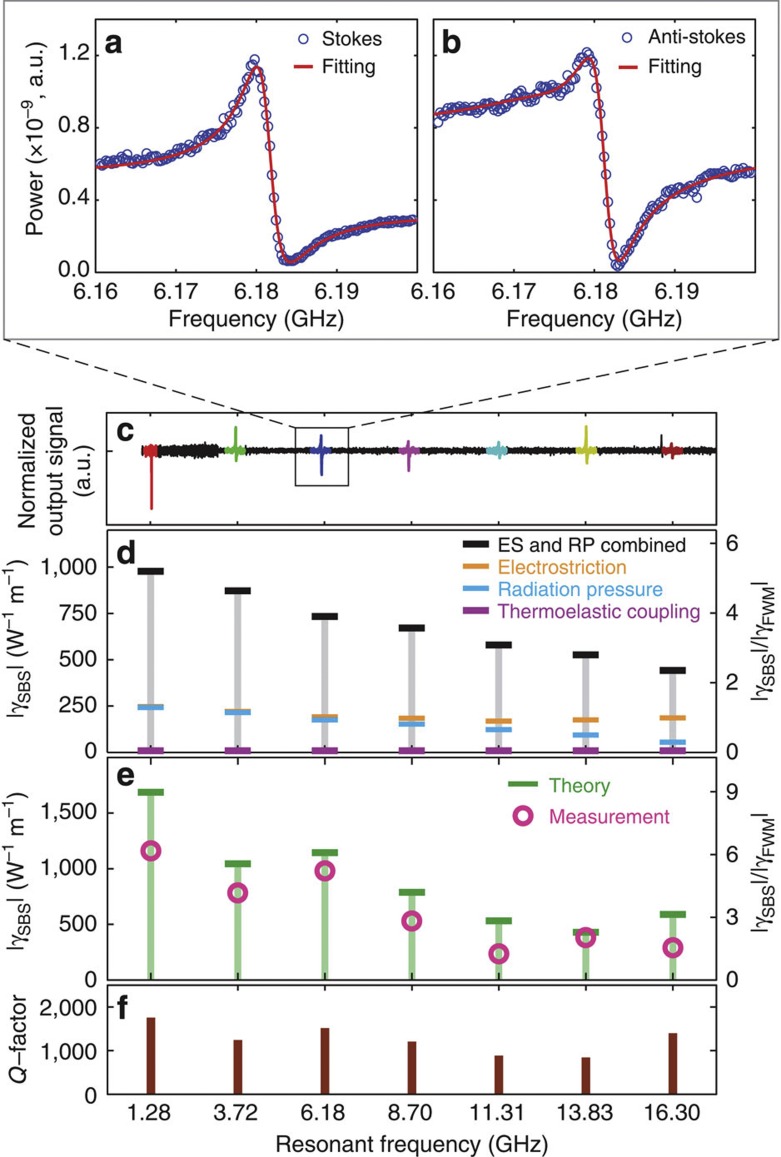
Characteristic spectral Brillouin line shapes and nonlinear coefficients obtained from both experiments and simulations. (**a**,**b**) Spectrally resolved Stokes and anti-Stokes Brillouin lines shapes, respectively, for the *m*=3 resonance of *w*=3.8 μm BAM waveguide. The theoretical fit (red line) obtained using the line shape derived in Supplementary Note 2 are shown as red curves atop the experimental data (circles). (**c**) Normalized heterodyne signals measured by the RF power meter for *w*=3.8 μm. (**d**) Simulated contributions of radiation pressure (blue bar), ES forces (orange bar) and thermoelastic coupling (violet bar) to the total Brillouin non-linear coefficient (black bar) are numerically calculated for *w*=3.8 μm for a fixed phononic *Q* of 1,000. (**e**) Comparison between the experimentally obtained (pink circle) and the theoretically calculated (green bar) SBS gain coefficients when experimentally measured quality factor of each resonant mode is incorporated within simulations. (**f**) Measured *Q*-factor of each resonant mode versus frequency for each resonance of the *w*=3.8 μm BAM waveguide, corresponding to modes *m*=1,2,…7 identified in Fig. 2.

Close examination of the resonance signatures in Fig. 4 reveals a Fano-like line shape produced by each Brillouin resonance, from which the magnitude of the Brillouin non-linear coefficient, *γ*_SBS_, can be obtained. This line shape can be more clearly seen from the high-resolution spectral scans of Fig. 5a, which show the line shape of a characteristic Brillouin resonance (*f*=6.185 GHz, with *w*=3.8 μm) decomposed into its Stokes and anti-Stokes components. These data were obtained by spectrally resolving the distinct heterodyne tones of the Stokes and anti-Stokes signals using a high-resolution RF spectrum analyser (SA) as the pump modulation frequency was swept. This asymmetric line shape results from the coherent interference between the Brillouin and electronic Kerr non-linearities of the waveguide. Involvement of electronic Kerr non-linearities at the Stokes and anti-Stokes frequencies occurs due to cross-phase modulation between the pump and probe beams within the silicon waveguide core. Note that the fibre apparatus yields a negligible contribution to the measured Brillouin and Kerr non-linearities of the device.

To precisely determine the magnitude of the Brillouin non-linear coefficient, *γ*_SBS_, relative to the intrinsic Kerr non-linear coefficient *γ*_K_ and the non-linear free-carrier dispersion coefficient *γ*_FC_ from these data, the non-linear coupled amplitude equations were formulated to derive the functional form of the Stokes and anti-Stokes line shapes (see Supplementary Note 2). As SBS is a resonant effect, its non-linear coefficient takes on a Lorentzian line shape centred about each Brillouin-active phonon mode. In contrast, the electronic Kerr non-linearities are non-resonant at 1,550 nm wavelengths, yielding a frequency-independent non-linear coefficient. As in the fibre-based studies of Wang *et al*.^40^, the frequency-dependent interference between the Kerr and Brillouin effects produces the asymmetric (Fano-like) line shape observed in Fig. 5a. However, it should be noted that our experimental arrangement is distinct, leading to a different set of coupled amplitude equations. In addition, non-linearly generated free carriers in silicon are responsible for the dissimilar line shapes of the Stokes and anti-Stokes orders, and a larger non-linear background for frequencies below 2 GHz under our experimental conditions. As the free-carrier effects roll off at high frequency, the Kerr responses at 16 GHz are used as a reference to determine the magnitude of the Brillouin non-linear coefficient, similar to that in ref. 52.

On the basis of the coupled amplitude model described in the Supplementary Note 2, the magnitude of the Brillouin non-linear coefficient, *γ*_SBS_, is extracted from the experimental line shape of both the Stokes and anti-Stokes signatures of each resonance of the *w*=3.8 μm BAM waveguide. Seven resonances, spanning frequencies from 1.28 to 16.30 GHz, are shown in Fig. 5c. The peak value of |*γ*_SBS_|/|*γ*_K_| and the phononic *Q*-factor of each resonance extracted from experiments (including separately resolved Stokes and anti-Stokes signatures) are shown Fig. 5e respectively. The peak value of the Brillouin non-linear coefficient at 1.28 GHz is found to be 6.18 times larger than the Kerr non-linear coefficient of the waveguide (or |*γ*_SBS_|/|*γ*_K_|=6.18). From the established non-linearities of silicon^53^, |*γ*_K_| of 188±34 W^−1^ m^−1^ was found for BAM waveguides with *w*=[1.8, 2.8, 3.8] μm (for further details see Supplementary Note 2)^54^,^55^. From this relative measurement, the Brillouin non-linear coefficient is found to be 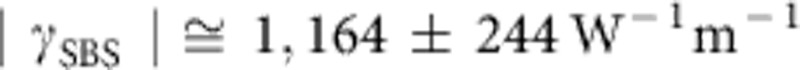 over the Brillouin-active region of the BAM waveguide. Moreover, as the Brillouin non-linear coefficient is related to the Brillouin gain as 2|*γ*_SBS_|=*G*_SBS_, this non-linearity corresponds to a forward SBS gain of 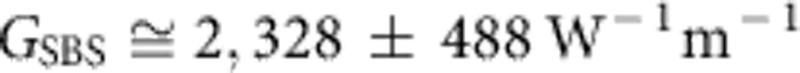. Note that much of the ±18% and ±21% uncertainty assigned to |*γ*_K_| and |*γ*_SBS_| values, respectively, arise from the (±15%) uncertainty in the measured value of the Kerr non-linearity of silicon^53^. Despite the fact that this non-linear response is the aggregate of an ensemble of 26 distinct Brillouin-active suspended regions fabricated along the length of the waveguide, remarkably high mechanical *Q*-factors (~1,000) are produced for phonon frequencies of 1.28 to 16.3 GHz.

### Comparison with theory

For comparison with experiments, full-vectorial three-dimensional multi-physics simulations were performed through coupled optical force and elastic wave COMSOL models following the approach outlined in ref. 41. The distinct contributions of ES forces (orange), radiation pressure (blue) and thermoelastic expansion (violet) to the total SBS nonlinear coefficient (black) are shown in Fig. 5d for each phonon resonance. Here a fixed mechanical *Q*-factor of *Q*=1,000 is assumed. Note that negligible contribution to the Brillouin coupling is produced by thermoelastic response at these GHz frequencies due to the slow thermal time constant of this system (for further details, see the Supplementary Discussion). The magnitude of the Brillouin coefficient, |*γ*_SBS_|, scales quadratically with optical force, yielding a non-linear addition of the radiation pressure and electrostrictively induced couplings to the overall Brillouin gain as seen in Fig. 5d (ref. 41). The total Brillouin non-linearity, which is almost exclusively driven by radiation pressure and electrostriction, slowly decreases with increasing resonant frequency as seen in Fig. 5d. A larger variation in Brillouin non-linearity is seen from the experimental data (circles of Fig. 5e) than from simulations (black bars of Fig. 5d) due to the variation of the measured phononic *Q* with frequency (Fig. 5f). However, when the frequency dependence of measured *Q*-factors is included in simulations (Fig. 5f), good agreement between simulations and experiments are obtained over the entire frequency range (green bars of Fig. 5e).

Both the highly localized electrostriction and radiation pressure force distributions within the waveguide core yield a frequency-dependent Brillouin gain (seen in Fig. 5d) exhibiting a significant departure from conventional backwards SBS processes involving bulk acoustic waves. In contrast to the rapid 1/Ω roll off of Brillouin gain with phonon frequency found through backward SBS^41^,^56^,^57^, the experimental (and simulated) *Q*-factor normalized Brillouin coefficient varies by less than 40% in magnitude over the entire 1–16 GHz frequency range. Unlike conventional systems where the overlap between the optical force distribution and the phonon mode profile is largely frequency independent, the complex double-lobed spatial force distributions in the core of the silicon waveguide produce a frequency-dependent overlap with various phonon modes, reshaping the frequency dependence of Brillouin coupling. The effect of spatial force distribution on the frequency dependence of coupling can be clearly seen by comparing the computed contributions of electrostriction and radiation pressure to the Brillouin gain of Fig. 5d. Although the radiation pressure contribution diminishes quite rapidly with frequency, the ES component varies by only a few per cent over 1–16 GHz frequency range. The higher bandwidth of ES coupling results from the higher spatial frequencies of the ES force distribution. Consequently, the relatively flat Brillouin gain produces efficient photon–phonon coupling over an unprecedented frequency range. The magnitude and frequency dependence of the measured Brillouin coupling, and their good agreement with simulations, all provide strong evidence of the important role of both electrostriction and radiation pressure within these BAM waveguides.

### Stimulated Brillouin-scattering gain measurement

**Figure 6:**
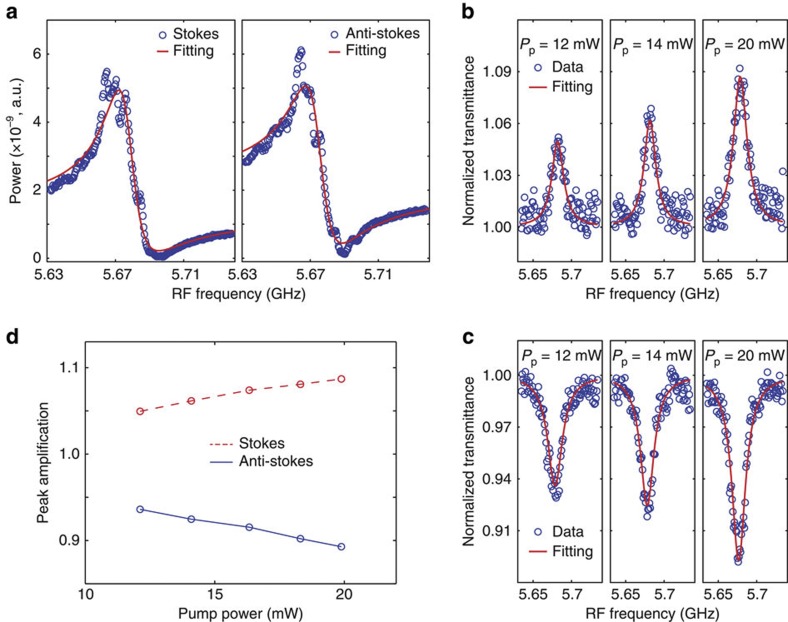
Normalized transmittances of Stokes and anti-Stokes Brillouin signals and characteristic spectral Brillouin line shapes. (**a**) Spectrally resolved Stokes and anti-Stokes Brillouin lines shapes, respectively, obtained through heterodyne FWM experiments. (**b**,**c**) Normalized transmittances of Stokes and anti-Stokes Brillouin signals, respectively, for the *m*=1 resonance of *d*=0.8 μm BAM waveguide for the pump powers of 12, 14 and 20 mW. (**d**) Peak amplification (absorption) of Stokes (anti-Stokes) signals versus pump power obtained from the line shapes of **b** and **c**.

The series of Brillouin resonances generated by the *w*=3.8 μm device provide insight into the bandwidth and frequency dependence of the Brillouin coupling, though a larger overall Brillouin non-linearity can be achieved with a higher degree of phonon confinement (that is, smaller values of *w*). Figure 6a shows the Stokes and anti-Stokes spectral line shapes obtained by through-measurement of the *w*=0.8 μm BAM waveguide device. In contrast to the *w*=3.8 μm device, several sharp spectral features, consistent with high *Q*-factor (*Q~*1,500) phononic resonances, are observed within the central Brillouin line shape, suggesting significant inhomogeneous broadening due fabrication non-uniformities in this case. A fit of the aggregate Brillouin line shape using a single Lorentzian oscillator model yields is seen in Fig. 6a, yielding *G*_SBS_ and *Q*-values of 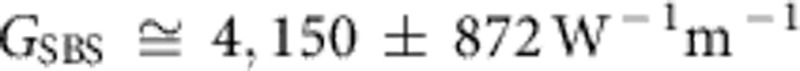 and *Q*
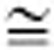
280. However, uncertainty in the form of inhomogeneously broadened line shape made it difficult to obtain a high confidence estimate of the Brillouin non-linearity in this case, prompting us to explore Brillouin non-linearities through direct measurement of Brillouin gain.

**Figure 7:**
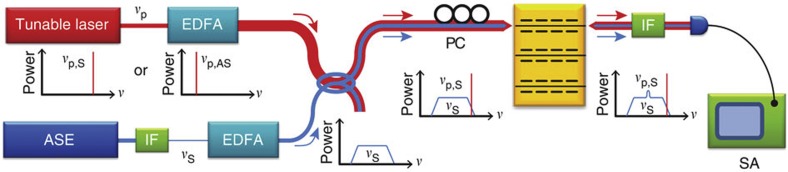
Experimental gain measurement apparatus used to measure the Brillouin gain spectrum of the BAM waveguide. ASE signal (*ν*_s_) beam is combined with the pump (*ν*_p_) beam, and both beams are coupled into and out of the waveguide with *d*=0.8 μm using lensed fibres. Two identical interference filters (IFs) are used to create unbalanced input/output signals. The pump frequency is located at the 3-dB band edge of the IFs, higher frequency for measuring the Stokes signal and lower frequency for the anti-Stokes signal. The strong pump beam induces the SBS gain at resonant frequencies, and the interference signal between the pump and ASE fields is monitored by an RF spectrum analyser (SA). PC, polarization controller; EDFA, erbium-doped fibre amplifier.

Experimental studies of Brillouin gain, performed by injecting strong pump and weak signal fields into a Brillouin-active waveguide (*w*=0.8 μm), are shown in Fig. 7. A low spectral-intensity amplified spontaneous emission (ASE) probe signal (centre wavelength: 1,552.94 nm; bandwidth: 50 GHz) was used in conjunction with a high-intensity pump laser (*λ*_p,S_=1,552.723 nm or *λ*_p,AS_=1,553.158 nm) to perform Brillouin gain measurements about the Stokes and anti-Stokes frequencies, respectively. The spectral power density of the ASE signal beam was measured by monitoring the heterodyne interference between the pump and signal fields with a receiver and an RF SA for frequencies about the Brillouin resonance (*v*=5.68 GHz).

The transmitted ASE power spectral density for higher pump powers are normalized to the power spectral density at lower pump powers (2.6 mW) to observe the power-dependent form of the Stokes and anti-Stokes line shapes. The normalized Stokes and anti-Stokes transmittances for *P*_p_=12, 14 and 20 mW are shown in Fig. 6b, respectively. The finer structure produced by inhomogeneous broadening is not visible because of the smaller signal-to-noise ratio obtained by this method. Lorentzian fits of the Stokes and anti-Stokes line shapes yield a *Q*-factor of ~300, indicating significant inhomogeneous broadening in this case. As the anti-Stokes process involves transfer of energy from the signal to the pump beam, the anti-Stokes signature (Fig. 6c) exhibits depletion instead of gain as shown in Fig. 6b. The theoretical fits (red line) performed using the line shape derived in Supplementary Note 3 are shown as red curves atop the experimental data (blue circles). In the small signal limit, which we explore through these experiments, the SBS gain is proportional to the pump power, and the amplification (and depletion) at resonant centre frequency is linear with the pump power as shown in Fig. 6d. A total effective forward SBS gain of 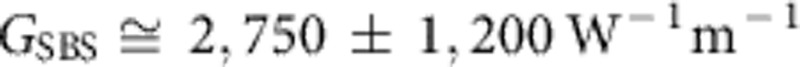 was extracted by fitting the data in Fig. 6b as described in the Supplementary Note 3. This measurement also shows good agreement with the simulated value of Brillouin gain, 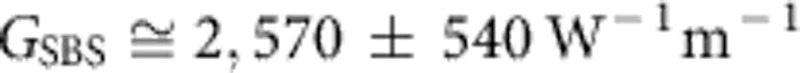. Note that these values of Brillouin gain are more than a factor of 10 larger than those obtained by treating the Brillouin non-linearities of silicon as a bulk medium property, providing strong evidence of the role of boundaries in shaping non-linearity at subwavelength scales. These measurements clearly demonstrate a gain coefficient that is over 1,000 times larger than recent demonstrations of forward SBS in fibres^12^, and several times larger than the Raman gain produced by silicon^19^,^21^, making Brillouin non-linearities the dominant third-order non-linearity in these silicon waveguides.

## Discussion

Although slight inhomogeneous broadening due to device dimension variability is observable from some of the measured Brillouin line shapes of various devices, consistent Brillouin gain and high *Q*-factor Brillouin resonances are found across the fabricated wafers. The nearly frequency-independent *Q*-factors observed through experiments suggest that factors other than intrinsic material dissipation limit the *Q*-factors observed here^58^,^59^. Hence, significant further enhancements in *Q*-factor and Brillouin gain should be attainable with device refinement and tighter dimensional control.

In conclusion, we have made the first demonstration of travelling-wave Brillouin non-linearities and Brillouin gain in silicon waveguides through a novel class of hybrid photonic–phononic waveguides. Through quantitative measurements, forward SBS non-linear susceptibilities were measured to be more than 1,000 times stronger than any previous waveguide system. Multi-physics simulations reveal that this strong photon–phonon coupling is produced by a constructive combination of ES forces and radiation pressures at nanoscales. The emergence of large radiation pressure-induced couplings represents a new form of boundary-induced Brillouin non-linearity^41^,^48^,^49^ and a new regime of boundary-mediated Brillouin coupling that arises in subwavelength structures. This novel waveguide geometry enables independent control of phononic modes and optomechanical driving forces to yield tailorable Brillouin coupling over exceptionally wide bandwidths. Simultaneous coupling to numerous transverse phonon modes yields a relatively flat Brillouin gain over this entire 1–18 GHz frequency range. We have demonstrated that structural tuning of phononic resonances from 1 to 18 GHz with high-quality factor (>1,000) yields tailorable non-linear optical susceptibilities due to the coherent interference of Kerr and Brillouin effects. In this system, the wideband nature of this photon–phonon coupling results from the highly localized optical forces produced within the nanoscale waveguide. These wideband and high-frequency (18 GHz) characteristics were achieved without the need for ultra-high-resolution lithography, significantly extending the frequency range of chip-scale photon–phonon coupling over state-of-the-art cavity optomechanical technologies.

Efficient coupling between a continuum of optical and phononic modes through such chip-scale travelling-wave Brillouin processes opens up a host of wideband signal-processing capabilities with CMOS-compatible silicon photonics, including pulse compression^13^, pulse and waveform synthesis^12^,^14^,^15^,^44^, coherent frequency comb generation^12^,^15^,^16^, variable bandwidth optical amplifiers and filters^44^,^45^,^46^, and coherent beam-combining schemes^47^. Travelling-wave Brillouin non-linearities can also produce optical phase conjugation^60^ and opto-acoustic isolators^18^ that are necessary to reduce signal distortions and eliminate parasitic reflections on silicon chips. In addition, the highly controllable nature of the phonons emitted by this hybrid photonic–phononic system could enable forms of coherent information transduction through travelling-wave processes that are complementary to recent cavity optomechanical systems. As efficient Brillouin-based photon–phonon conversion is possible over wide bandwidths (>20 GHz), and the Brillouin-emitted phonons can be guided and manipulated on chip, hybridization of Brillouin device physics with silicon photonics, CMOS and microelectromechanical systems could provide a host of new coherent signal-processing technologies.

## Methods

### Fabrication methods

The silicon waveguides were patterned in a silicon-on-insulator with at 3,000 nm oxide undercladding using an ASML deep UV scanner, and etched in an AMAT DPS polysilicon etch tool. Following resist strip and standard post-etch and pre-diffusion cleans, LPCVD Si_3_N_4_ of 300 nm thickness was deposited in an SVG series 6000 vertical. A chemical–mechanical polish was used to preferentially thin conformal nitride atop Si waveguide. Hot phosphoric acid etch was used to clear the remaining nitride atop the silicon waveguide. The net result is the waveguide cross-section seen in Fig. 1. The nitride layer was then patterned to form the air slots seen in Fig. 1. Facet cuts for fibre access were created by patterning thick resist using a 1 × mask in a SUSS MA-6 contact aligner and employing deep-RIE etch. The oxide undercladding was then released in a 49% hydrofluoric acid etch.

### Experimental methods

The pump beam (at 1,556 nm) is modulated using a Mach-Zehnder intensity modulator. Mutually incoherent light from another distributed feedback laser at 1,536 nm is used as the probe beam. The probe beam splits into two paths to form a heterodyne interferometer. In the upper arm of the interferometer, the probe beam is combined with the pump beam using a wavelength division multiplexer. Both pump and probe beams are coupled into and out of the waveguide using lensed fibres. The pump wave exiting the device is blocked by an interference filter such that no pump light could be detected. The probe beam is frequency shifted by Δ=−165 MHz using an acousto-optic modulator in the lower arm of the interferometer to form the local oscillator for heterodyne detection. In traversing the Brillouin device in the upper arm of the interferometer, the pump- beam produces non-linearly sidebands (or a signal) on the probe beam. At the output of the interferometer, local oscillator is then mixed with the probe beam and signal using a 30:70 directional coupler, and detected using a high-speed (18 GHz) receiver. The RF signals produced through detection are then measured with either an RF power meter or an RF spectrum analyser. Because of the −165 MHz frequency offset of the local oscillator, the Stokes and anti-Stokes signatures can be observed as separate tones in the RF SA, with a total frequency separation of 330 MHz. By scanning the drive frequency of the RF signal generator while measuring either RF power or RF spectrum, detailed analysis of the Brillouin response of the system is made. A fibre-to-chip coupling loss of 8 dB and waveguide propagation loss of 7 dB cm^−1^ were estimated through waveguide cutback measurements. Pump and probe powers internal to the waveguide are estimated to be 6.5 mW and 9.6 mW, respectively.

## Author contributions

All authors made important contributions to this work. R.J. and A.S. fabricated the waveguide devices. P.R. designed devices with assistance of R.H. and Z.W. W.Q. and Z.W. developed multi-physics simulations. W.Q., Z.W., J.C. and P.R. performed numerous device simulations to understand various aspects of experiments. H.S., W.Q., Z.W. and P.R. developed analytical models to describe device non-linearities and Brillouin coupling. H.S., J.C. and P.R. developed the experimental techniques. H.S. conducted experiments with assistance of P.R. H.S., Z.W. and P.R. conceived and designed the experiments. All authors contributed to the writing of this paper.

## Additional information

**How to cite this article:** Shin, H. *et al*. Tailorable stimulated Brillouin scattering in nanoscale silicon waveguides. *Nat. Commun.* 4:1944 doi: 10.1038/ncomms2943 (2013).

## Supplementary Material

Supplementary Information Supplementary Figure S1, Supplementary Notes 1-3, Supplementary Discussion and Supplementary References
