# Supplementary material for: Tailorable stimulated Brillouin scattering in nanoscale silicon waveguides
Source: Nat Commun. 2013 Jun 6;4:1944. doi: 10.1038/ncomms2943 (PMC3709496; doi:10.1038/ncomms2943)
Supplement: Supplementary Information — Supplementary Figure S1, Supplementary Notes 1-3, Supplementary Discussion and Supplementary References [file ncomms2943-s1.pdf]

Tailorable stimulated Brillouin scattering in nanoscale silicon waveguides.

Heedeuk Shin[1], Wenjun Qiu[2], Robert Jarecki[1], Jonathan A. Cox[1], Roy H. Olsson III[1], Andrew Starbuck[1], Zheng Wang[3], and Peter T. Rakich[4*]

[1]Sandia National Laboratories, PO Box 5800 Albuquerque, NM 87185 USA
[2]Department of Physics, Massachusetts Institute of Technology, Cambridge, MA 02139 USA
[3]Department of Electrical and Computer Engineering, University of Texas at Austin, Austin, TX 78758 USA
[4]Department of Applied Physics, Yale University, New Haven, CT 06520 USA

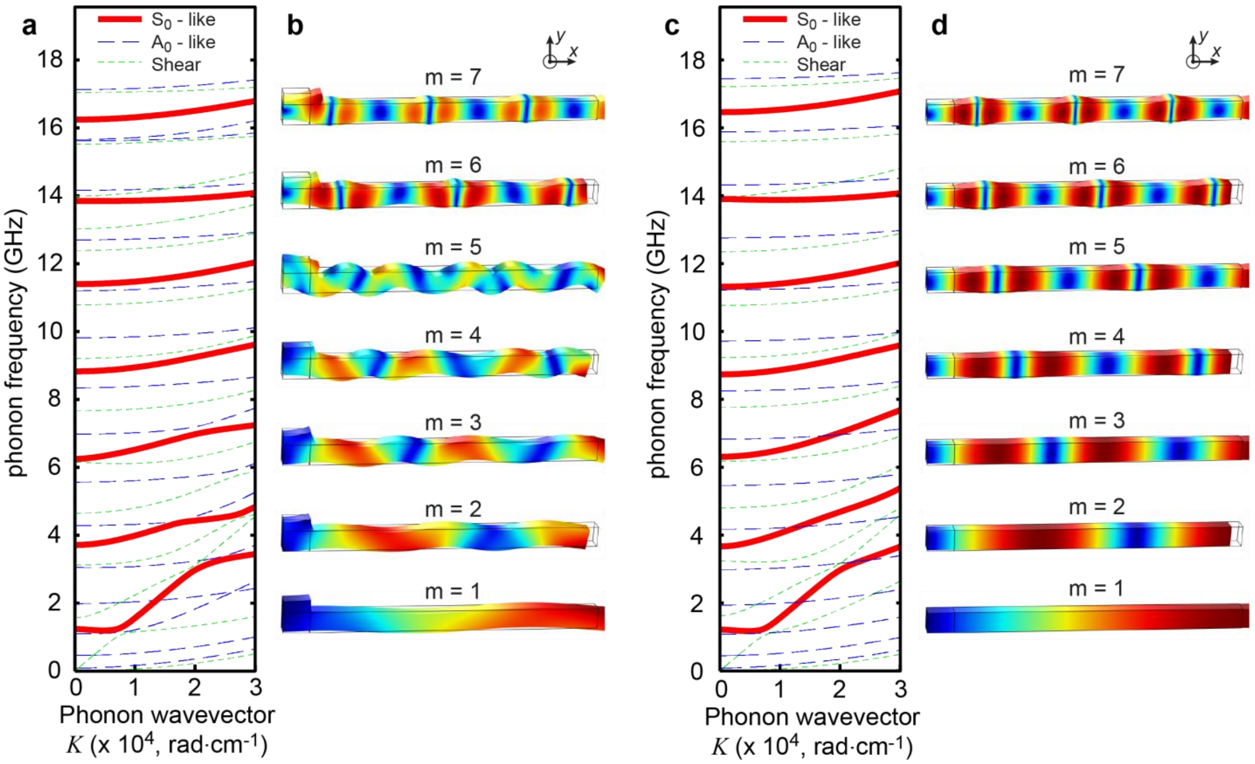

Supplementary Figure S1| Phononic dispersion and elastic wave displacements produced by two different BAM-waveguide devices. (**a**,**b**) **a** and **b** show the phononic dispersion and displacement fields respectively of the BAM waveguide studied in experiments. (**c**,**d**) **c** and **d** show the phononic dispersion and displacement fields respectively of an idealized BAM-waveguide possessing vertical symmetry.

Supplementary Note 1

Classification of Brillouin-Active Guided Phonons.

The numerically computed Brillouin-active guided phonon modes within the fabricated BAM-waveguide ($\boldsymbol{w = 3.8\ \mu m}$) are shown in Supplementary Figure S1 for comparison with a similar idealized BAM-waveguide possessing vertical symmetry. Through finite element models, symmetric boundary conditions were applied to the left-most domain boundary of the bisected BAM-waveguide segments seen in Supplementary Figure S1b and S1d, limiting the computed modes to those possessing even symmetry about to the silicon waveguide core. Periodic boundary conditions are applied to the z-normal faces of this simulation domain to compute the displacement fields of the phase matched phonon modes in Supplementary Figure S1b and S1d. The phonon dispersion curves in Supplementary Figure S1a and S1c were computed by varying the longitudinal wave-vector ($\mathbf{K}$) associated with the periodic boundary condition. Note that the Brillouin coupling is limited to phonons with symmetric displacement fields about the waveguide core since the TE-like mode considered here produces optical force distributions are symmetric with respect to the centre of the silicon waveguide core. Supplementary Figure S1a and 1c show the dispersion of the extensional ($\boldsymbol{S_0}$-like), flexural ($\mathbf{A_0}$-like) and shear waves of the system. Here, $\boldsymbol{S_0}$ represents the fundamental symmetric Lamb-wave, which is compressive in character, and $\mathbf{A_o}$ is the asymmetric fundamental Lamb-wave.

The Brillouin-active phonon modes under study, (i.e., those exhibiting good overlap with the optical force) are shown as solid red curves in Supplementary Figure S1a and 1c, alongside the computed elastic displacement fields. While the displacement fields of Supplementary Figure S1b exhibit some flexural character, the modes of Supplementary Figure S1d show only longitudinal compressive motion, consistent with symmetric Lamb-waves ($\boldsymbol{S_0}$). Despite this flexural character induced by the vertical asymmetry, a comparison of the dispersion curves of Supplementary Figure S1a and S1c clearly reveal that the phonon modes examined in our experimental system are symmetric Lamb-waves. Owing to their low phononic nonlinearities,

low signal distortion, and their linear phononic dispersion, symmetric Lamb-waves are routinely used for signal transduction in MEMS.

Supplementary Note 2

Brillouin resonance with Kerr nonlinearities.

Next, we develop the coupled wave equations which describe the nonlinear wave-mixing processes within our Brillouin waveguides, and derive functional form of the asymmetric line-shapes observed through heterodyne pump-probe experiments. Using the analytically derived line-shapes, quantitative analyses of the experimental signatures are performed to determine the magnitude of the Brillouin nonlinear coefficient.

Through experimental arrangement described in the body of this manuscript mutually incoherent pump and probe beams are coupled into the Brillouin waveguide. The pump beam is produced by intensity modulation of the monochromatic laser line. Modulation at frequency Ω generates a pump beam consisting of two frequencies ω_1 and ω_2 with corresponding wave-amplitudes A_1 and A_2, where $\omega_2 - \omega_1 = \Omega$. The probe beam consists of a monochromatic wave of disparate wavelength, with wave amplitude A_3 and frequency ω_3. Nonlinear wave-mixing processes involving A_1, A_2 and A_3 generate Stokes and anti-Stokes fields at frequencies $\omega_\mathrm{s} = \omega_3 - \Omega$, and $\omega_\mathrm{a} = \omega_3 + \Omega$, with wave amplitudes A_s and A_a, respectively. These A_s and A_a wave-amplitudes are measured through heterodyne detection to produce the line-shapes discussed in the body of this manuscript. For simplicity, we assume that both pump and probe waves are coupled to TE-like waveguide mode with wave powers defined as $P_i = |A_i|^2$. Since the Stokes and anti-Stokes waves have zero amplitude at the waveguide entrance, the coupled wave equations for Stokes and anti-Stokes wave growth can, to first order, be expressed as[61,62,63],

$$\frac{dA_\mathrm{s}}{dz} = i\left[\gamma_\mathrm{SBS}^{(3)^*}(\Omega) + 2\gamma_\mathrm{K}^{(3)} + \gamma_\mathrm{FC}^{(5)}(-\Omega)P_0\right] A_1^* A_2 A_3 \tag{S1a}$$

$$\frac{dA_\mathrm{a}}{dz} = i\left[\gamma_\mathrm{SBS}^{(3)}(\Omega) + 2\gamma_\mathrm{K}^{(3)} + \gamma_\mathrm{FC}^{(5)}(+\Omega)P_0\right] A_1 A_2^* A_3 \,. \tag{S1b}$$

Here, $P_0 = 2(|A_1|^2 + |A_2|^2 + |A_3|^2)$, and $\gamma_{\mathrm{SBS}}^{(3)}(\Omega)$ and $\gamma_{\mathrm{K}}^{(3)}$ are the third order nonlinear coefficients for Brillouin scattering (SBS) and non-degenerate four-wave mixing (FWM), respectively. For simplicity, we have also neglected two-photon absorption (TPA) induced attenuation of A_{s} and A_{p}, since in this small signal limit, these terms are much smaller than the source terms of Eq. (S1a) and Eq. (S1b). We assume that the Brillouin nonlinearity, $\gamma_{\mathrm{SBS}}^{(3)}(\Omega)$, is described by a single oscillator, yielding a Lorentzian line-shape of the form[61],

$$\gamma_{\mathrm{SBS}}^{(3)}(\Omega) = \frac{G}{2}\frac{\Omega_m/2Q}{\Omega_m - \Omega - i\,\Omega_m/2Q}. \tag{S2}$$

Above, Ω_m is the resonant frequency of the mth mode, Q indicates the quality factor of the phonon resonator, and $G = 2\left|\gamma_{\mathrm{SBS}}^{(3)}(\Omega_m)\right|$ is the Brillouin gain. In addition, $\gamma_{\mathrm{FC}}^{(5)}(\Omega)$ is the fifth order nonlinear coefficient which results from two-photon absorption (TPA) induced the free carrier absorption and refractive index changes imparted by waves A_1, A_2 and A_3. Solving for time-harmonically modulated TPA-induced free carrier generation rate[63], and using the carrier rate equation to solve for $\gamma_{\mathrm{FC}}^{(5)}(\Omega)$, one finds,

$$\gamma_{\mathrm{FC}}^{(5)}(\pm\Omega) \equiv -\left(\frac{M}{\tau} \pm \frac{\mathrm{V}\Omega}{2}\right)\frac{1}{1/\tau^2 + \Omega^2} . \tag{S3}$$

Here M and V are constants with positive value, and τ is the free carrier lifetime. Note that $\gamma_{\mathrm{K}}^{(3)}$ is, to an excellent degree, described as a frequency independent constant which is computed from the waveguide geometry and the nonlinear coefficient of silicon following Refs [54,55]. Thus, Kerr nonlinearities are non-dispersive, while Brillouin and the free carrier induced nonlinear couplings have frequency dependent responses in our frequency sweeping range. To remain consistent with our experimental arrangement, we note that the FWM and free-carrier effect occur through the waveguide entire waveguide length (4.9 mm), while the Brillouin-active interaction length is shorter than the total waveguide length (2.6 mm). In this case, the optical power of the Stokes field obtained by solving equation (S1a) is given by,

$$\mathrm{g_s} = C\left|\gamma_{\mathrm{SBS}}^{(3)^*}(\Omega)L_{\mathrm{SBS}} + \left(2\gamma_{\mathrm{K}}^{(3)} + \gamma_{\mathrm{FC}}^{(5)}(-\Omega)P_0\right)L_{\mathrm{tot}}\right|^2 P_1P_2P_3 . \tag{S4}$$

where C is a constant, P_k indicates the optical power of kth field, and L_{SBS} and L_{tot} are the interaction lengths of SBS and the rest nonlinear responses, respectively. Equation (S4) consists of two terms, one for Brillouin scattering and another which includes both non-degenerate four-wave mixing and free carrier effects. We refer to the background signal produced by FWM and free carrier effect as the reference signal, as these are present within the reference silicon waveguides. In the absence of the Brillouin nonlinearities (e.g. for large detuning from a Brillouin resonance) the free carrier and FWM contributions to the Stokes sideband can be described as,

$$\mathrm{g_{os}} \equiv C L_{\text{tot}}^2 \left| 2\gamma_{\mathrm{K}}^{(3)} + \gamma_{\mathrm{FC}}^{(5)}(-\Omega) P_0 \right|^2 P_1 P_2 P_3. \qquad \text{(S5)}$$

Since the free carrier effects has slowly varying envelope in frequency, $\gamma_{\mathrm{FC}}^{(5)}(\Omega)$ can be treated as a constant in the vicinity of a single Brillouin resonance (e.g. for frequency spans of less than 100 MHz). The functional form of $\gamma_{\mathrm{FC}}^{(5)}(\Omega)$, seen from Eq. (S3), reveals that $\gamma_{\mathrm{FC}}^{(5)}(-\Omega) \neq \gamma_{\mathrm{FC}}^{(5)}(\Omega)$. Hence, $\gamma_{\mathrm{FC}}^{(5)}$, or nonlinear background, takes on a different value at Stokes and anti-Stokes frequencies.

By fitting equation (S4) to the experimentally obtained Stokes and anti-Stokes Brillouin scattering signals as shown in Fig. 5a-b, we can estimate the Brillouin gain $G = 2\left|\gamma_{\mathrm{SBS}}^{(3)}(\Omega_m)\right|$. The normalized fitting function $\mathrm{g_s}/\mathrm{g_{os}}$ is derived from Eq. (S4) and (S5) as,

$$\frac{\mathrm{g_s}}{\mathrm{g_{os}}} = \left| e^{ib_{\mathrm{S}}} + D_m \frac{\Omega_m/2Q}{\Omega_m - \Omega - i\,\Omega_m/2Q} \right|^2, \qquad \text{(S6)}$$

where $D_m \equiv G L_{\text{SBS}} / \left(2 L_{\text{tot}} \left| 2\gamma_{\mathrm{K}}^{(3)} + \gamma_{\mathrm{FC}}^{(5)}(-\Omega) P_0 \right| \right)$ is the relative strength of the Brillouin scattering effect relative to the reference nonlinear responses. Because $\gamma_{\mathrm{SBS}}^{(3)}(\Omega)$ and $\gamma_{\mathrm{FC}}^{(5)}(\Omega)$ are complex functions, the relative phase between the Brillouin scattering signal and background (FWM and FC) nonlinear responses is defined as b_{S} in equation (S6). The proportionality to P_1, P_2, and P_3 as well as the constant C in equations (S4) and (S5) are normalized out of equation (S6).

Note that because of the frequency dependent free-carrier effect, different resonant modes are normalized by different nonlinear background. In experiments, we observed that at high frequency (>15 GHz) the amplitude of the reference signal converges to $\left|2\gamma_{\mathrm{K}}^{(3)}\right|$, indicating $\left|2\gamma_{\mathrm{K}}^{(3)}\right| \gg \left|\gamma_{\mathrm{FC}}^{(5)}(\pm\Omega)P_0\right|$. Hence, we can experimentally measure the reference signal spectrum and obtain the ratio $\eta \equiv \left|2\gamma_{\mathrm{K}}^{(3)} + \gamma_{\mathrm{FC}}^{(5)}(\Omega_m)P_0\right| / \left|2\gamma_{\mathrm{K}}^{(3)}\right|$. Then using established methods to compute $\left|2\gamma_{\mathrm{K}}^{(3)}\right|$ based on established values for the Kerr nonlinearities of crystalline silicon [54,55], we can then estimate the Brillouin gain G using the following equation,

$$G = 2D_m\eta\left|2\gamma_{\mathrm{K}}^{(3)}\right|\frac{L_{\mathrm{tot}}}{L_{\mathrm{SBS}}}. \tag{S7}$$

Note that we have neglect propagation losses in (S1a) and (S1b), as losses do not alter the final functional form of the derived line-shape in the small signal limit.

The magnitude of $\gamma_{\mathrm{K}}^{(3)}$ produced by the silicon waveguide was computed using the accepted Kerr coefficient of $n_2 = 4.5 \times 10^{-18}\mathrm{m}^2\mathrm{W}^{-1}$ in silicon[63]. Employing the full-vectorial method for computing $\gamma_{\mathrm{K}}^{(3)}$ described in Ref. [54], $\left|\gamma_{\mathrm{K}}^{(3)}\right|$ was computed to be $188\ \mathrm{m}^{-1}\mathrm{W}^{-1}$ for TOPROW waveguides with nitride membrane widths of $w = [1.8,\ 2.8,\ 3.8]$ μm. As the nitride width was reduced to $w = 0.8$ μm, the close proximity of the lateral nitride boundary increases the modal confinement, yielding $\left|\gamma_{\mathrm{K}}^{(3)}\right|$ of $214\ \mathrm{W}^{-1}\mathrm{m}^{-1}$.

Supplementary Note 3

Analysis of the power-dependent Brillouin gain.

For simplicity, we begin by developing an analytical description of the Brillouin gain spectrum obtained through experiments in the absence of free-carrier and four-wave mixing induced nonlinearities. In this case, the coupled wave equations for Stokes and anti-Stokes wave growth in the gain spectrum method can be expressed as in Ref. [56],

$$\frac{dA_{\mathrm{s}}}{dz} = i\left[\gamma_{\mathrm{SBS}}^{(3)^*}(\Omega)\right]\left|A_{\mathrm{p}}\right|^2 A_{\mathrm{s}} \tag{S8a}$$

$$\frac{dA_{\mathrm{a}}}{dz} = i\left[\gamma_{\mathrm{SBS}}^{(3)}(\Omega)\right]\left|A_{\mathrm{p}}\right|^2 A_{\mathrm{a}} \tag{S8b}$$

and the power of Stokes wave varies as,

$$\frac{dP_{\mathrm{s}}}{dz} = G\frac{(\Omega_m/2Q)^2}{(\Omega_m-\Omega)^2+(\Omega_m/2Q)^2}P_{\mathrm{p}}P_{\mathrm{s}} \equiv gP_{\mathrm{p}}P_{\mathrm{s}} \tag{S9a}$$

$$\frac{dP_{\mathrm{a}}}{dz} = -G\frac{(\Omega_m/2Q)^2}{(\Omega_m-\Omega)^2+(\Omega_m/2Q)^2}P_{\mathrm{p}}P_{\mathrm{a}} \equiv -gP_{\mathrm{p}}P_{\mathrm{a}} \tag{S9b}$$

where P_{s} and P_{a} are the Stokes and anti-Stokes signal power with pump power of P_{p} at frequency $c/\lambda_{\mathrm{p,S}} - \Omega$ or $c/\lambda_{\mathrm{p,AS}} + \Omega$. g is the SBS gain factor forming a Lorentzian line shape, and G is the Brillouin gain. The solutions of these equations are given by

$$P_{\mathrm{s}}(\mathrm{L},\Omega,P_{\mathrm{p}}) = P_{\mathrm{s}}(0,\Omega,P_{\mathrm{p}})\exp(\mathrm{g}P_{\mathrm{p}}L_{\mathrm{eff}}) \tag{S11a}$$

$$P_{\mathrm{a}}(\mathrm{L},\Omega,P_{\mathrm{p}}) = P_{\mathrm{a}}(0,\Omega,P_{\mathrm{p}})\exp(-\mathrm{g}P_{\mathrm{p}}L_{\mathrm{eff}}) \tag{S11b}$$

where $L_{\mathrm{eff}} \equiv (1-\exp(-\alpha L))/\alpha$ is the effective propagation length of the waveguide. Experimentally, we measure the transmitted Stokes and anti-Stokes waves for different pump powers (P_{p}), and normalize the signal with one for the lowest pump power ($P_{\mathrm{p}} = 2.6\ \mathrm{mW}$). Therefore the fitting functions for Stokes and anti-Stokes gain are given by,

$$\frac{P_{\mathrm{s}}(L,\Omega,P_{\mathrm{p}})}{P_{\mathrm{s}}(L,\Omega,2.6\ \mathrm{mW})} = \exp\left[\left(G\frac{(\Omega_m/2Q)^2}{(\Omega_m-\Omega)^2+(\Omega_m/2Q)^2}\right)(P_{\mathrm{p}}-2.6\ \mathrm{mW})L_{\mathrm{eff}}\right] \tag{S12a}$$

$$\frac{P_{\mathrm{a}}(L,\Omega,P_{\mathrm{p}})}{P_{\mathrm{a}}(L,\Omega,2.6\ \mathrm{mW})} = \exp\left[-\left(G\frac{(\Omega_m/2Q)^2}{(\Omega_m-\Omega)^2+(\Omega_m/2Q)^2}\right)(P_{\mathrm{p}}-2.6\ \mathrm{mW})L_{\mathrm{eff}}\right]. \tag{S12b}$$

The estimated effective SBS propagation length of the waveguide under test is about 2.1 mm. As shown in Fig. 7**c**, a 10% differential-gain is produced at the Stokes wavelength for a change of pump power from 2.6 mW with 20 mW at resonant frequency ($\Omega - \Omega_m$). Hence, evaluation of the Brillouin gain using these numbers and Eq. (S12a), gives gain a value of approximately $2800\ \mathrm{W}^{-1}\mathrm{m}^{-1}$. Free carrier effects can be included in this analysis in a straightforward manner to develop the analogous expressions for line-shape and Brillouin gain. However, one can show that the inclusion of free carrier effects yields a small correction to the estimated Brillouin gain.

Supplementary Discussion

Brillouin nonlinearities were modeled within the TOPROW waveguides described in the body of the manuscript following the methods described in Refs. [41] and [50], using COMSOL finite element software. Electrostrictive forces were implemented based upon the measured photoelastic tensor coefficients of bulk crystalline silicon[64] with photoelastic constants of $[p_{11}, p_{12}, p_{44}] = [-0.094, +0.017, -0.051]$. Simulations assume crystallographic alignment of the waveguide axis with the [100] direction for both the tensor elastic and photoelastic properties of the system, which is consistent with the crystal orientation of the fabricated devices. No free parameters were used in the simulation results; all input parameters of the model (including dielectric constants, elastic constants, photoelastic constants, phononic Q-factor, and geometric properties) are derived directly from experimental measurement.

It should also be noted that thermoelastic coupling produces negligible contribution to the overall Brillouin coupling over all frequencies of interest. Transient thermal models reveal that the poor thermal conductivity of the nitride membrane (within which the silicon waveguide is embedded) produce a slow thermal response ($\tau \approx 20$ ns), limiting the bandwidth of thermoelastic coupling to ~ 8 MHz. Moreover, as compared to cavity optomechanical systems (having interaction lengths of 5-10 micron), the optically-induced temperature changes are much smaller within this waveguide. This is because heat absorption is distributed along the entire length of a 4.9 mm long waveguide. As a consequence, models of the thermoelastic coupling reveal that the thermoelastic driving forces are ~1000 times weaker than those of radiation pressure and electrostriction, yielding negligible contribution at these GHz frequencies in our waveguide device.

Supplementary References

61. R. W. Boyd, *Nonlinear Optics*, 3rd Ed. (Academic Press, San Diego, 2008).

62. A. Yariv, *Quantum Electronics*, 3rd Ed. (Wiley, 1989).

63. R. Dekker, N. Usechak, M. Först, and A. Driessen, *J. Phys. D: Appl. Phys.* **40**, R249 (2007).

64. D. Biegelsen, Phys. Rev. B, **32**, 1196 (1974).
